# Unexpectedly High and Difficult-to-Explain Regenerative Capacity in an 82-Year-Old Patient with Insulin-Requiring Type 2 Diabetes and End-Stage Renal Disease

**DOI:** 10.3390/jcm14082556

**Published:** 2025-04-08

**Authors:** Mihaela Gheorghiu, Maria-Florina Trandafir, Octavian Savu, Daniela Pasarica, Coralia Bleotu

**Affiliations:** 1 Pathophysiology and Immunology Department, “Carol Davila” University of Medicine and Pharmacy, 020021 Bucharest, Romania; maria.trandafir@umfcd.ro (M.-F.T.); octavian.savu@umfcd.ro (O.S.); daniela.pasarica@umfcd.ro (D.P.); 2 “N.C. Paulescu” National Institute of Diabetes, Nutrition and Metabolic Diseases, 020475 Bucharest, Romania; 3 Doctoral School of “Carol Davila” University of Medicine and Pharmacy, 020021 Bucharest, Romania; 4 “Stefan S. Nicolau” Institute of Virology, 030304 Bucharest, Romania; cbleotu@yahoo.com

**Keywords:** type 2 diabetes mellitus, end-stage renal disease, inflammatory cytokines, neurotrophin-3, vascular endothelial growth factor beta

## Abstract

**Background/Objectives:** The case we present is part of a large study that we conducted on hemodialysis patients with type 2 diabetes mellitus (T2DM) and which set the following objectives: studying changes in the intestinal microbiota, innate and acquired immune response capacity, and tissue regeneration. **Methods:** (1) For the genetic study of the gut microbiota, special techniques that are not based on cultivation were used since most of the species in the intestinal flora are not cultivable. (2) The immunological study had two targets: innate immunity (inflammation) and adaptive immunity (we chose to address the cellular immune response because, unlike the humoral one, it is insufficiently studied in this category of associated pathologies). As markers for innate immunity (inflammation), the following were determined: IL-6, sIL-6R, IL-1β, TNFα, IL-10, and NGAL. TNFβ/LTα was determined as a marker for adaptive immunity (the cellular immune response). (3) The study of tissue regeneration capacity was performed using NT-3 (this is the first study to do so) and VEGFβ (another marker that is scarce in this category of patients) as markers. All the aforementioned compounds were determined from serum samples, utilizing Merck Millipore ELISA kits for IL-6, IL-1β, IL-10, NT-3, and VEGF β, and Elabscience ELISA kits for IL-6R, TNFα, TNFβ, and NGAL. **Results:** We were very surprised to find unexpected immunological changes and tissue regenerative capacity in one of the patients studied, an 82-year-old female patient diagnosed with insulin-dependent T2DM with multiple complications, including end-stage renal disease (ESRD). The patient showed a huge capacity for tissue regeneration, combined with amplification of immunological capacity, in comparison to patients in the same group (T2DM and ESRD) and to those in the control group (ESRD). Thus, extremely elevated serum concentrations of IL-1β, IL-6, IL-10, and TNF-β, as well as the tissue regeneration indicators NT-3 and VEGFβ, were obtained in comparison to all other members of the patient group. At the same time, serum levels of the soluble IL-6 receptor (sIL6-R) and TNFα were greatly reduced compared to the test group’s mean. **Conclusions:** All the data obtained during our research were corroborated with those from the specialized literature and entitle us to support the hypothesis that the cause of these unexpected behaviors is the genetically conditioned overproduction (possibly acquired post-infection) of IL-6, along with its predominant anti-inflammatory and pro-regenerative signaling through the membrane-bound receptor IL-6R.

## 1. Introduction

Global statistics for 2023 indicated roughly 850 million cases of chronic kidney disease [1], of which more than 2.7 million require hemodialysis, a number that is expected to reach 5.4 million in 2030 [2]. In addition to the underlying disease that causes the need for chronic hemodialysis, these patients are also exposed to systemic stress, with an increased risk of cardiovascular, infectious, and neoplastic diseases [3]. A predisposition for these pathologies is induced by innate immunity (inflammation) activation and the senescence of adaptive immunity (humoral and cellular immune responses).

### 1.1. The Case

The case we set out to present surprised us with its immunological behavior and capacity for tissue regeneration, which are extremely atypical for the pathology it presented: an 82-year-old female patient diagnosed with ESRD (on hemodialysis), occurring as a complication of T2DM2. This case is part of a much more extensive research project, which included two groups of patients [4]. The test group consisted of T2DM patients with ESRD on hemodialysis, and the control group consisted of hemodialysis patients without diabetes. The 82-year-old patient S.A. was included in the test group and suffered from insulin-dependent T2DM with multiple complications (ESRD, diabetic polyneuropathy, and secondary hypertension). She also had NYHA class III heart failure, which was associated with a valvular disorder, degenerative aortic disease, ischemic coronary disease—ICD, stable angina pectoris, systemic atherosclerosis, chronic hepatitis C, cervical spondylosis, Parkinsonian syndrome, right femoral post-thrombotic syndrome, a significant insufficiency of the femoral, popliteal, and great saphenous veins, and superficial venous dilations on the medial calf. At the time of sample collection, she was in good general condition, lucid, and hemodynamically balanced.

She also did not meet the exclusion criteria of this study. She did not present with anemia from a non-renal cause, acute bleeding or a history of bleeding in the last 3 months, blood transfusions in the last 3 months, acute inflammation or infectious diseases, acute vascular pathology, or current treatment with immunosuppressors.

The treatment she was receiving at the time consisted of: amlodipine 5 mg ½ tablet × 2 times/day, atorvastatin 20 mg 1 tablet/day, salicylic acid 75 mg 1 tablet/day, folic acid 5 mg 1 tablet/day, Essentiale 1 tablet/day, Mixtard insulin as needed, pentoxifylline 400 mg 1 tablet/day, Neuromultivit 1 tablet/day, and pantoprazole 40 mg 1 tablet/day.

### 1.2. General Research Plan

The patient groups included in this study were investigated according to three separate research directions:

- the genetic study of the intestinal microbiota, in order to identify possible microbial alterations and their impact on the immunological behavior and underlying pathology of the patients;
- the complex study of innate immunity (inflammation) and acquired immunity (RIC);
- the study of tissue regeneration capacity.

## 2. Methods

### 2.1. The Genetic Study of the Gut Microbiota

Special techniques that are not based on cultivation were used since most of the species in the intestinal flora are not cultivable [5,6].

Bacterial DNA was extracted from stool samples using a commercial kit (Qiagen Stool Mini Kit; Qiagen, Inc., Germany, EU). The DNA concentrations were quantified utilizing the Qubit 4 fluorometer (Thermo Fisher Scientific, Inc., MA, USA). In order to investigate the gut microbiota, 16S ribosomal RNA (rRNA) and 18S rRNA primers were used and quantitative PCR was performed. For the quantification of the various bacterial and fungal populations, the following were utilized: 9 ng DNA isolated from the stool samples, SYBR Green 2X amplification kits (Applied Biosystems; Thermo Fisher Scientific, Inc., MA, USA), and 16S/18S rRNA primers (2.5 nM). Amplification was performed on a Viia7 instrument (Applied Biosystems; Thermo Fisher Scientific, Inc.). Each sample was analyzed in triplicate. Samples without a DNA template served as negative controls. Samples were incubated at 95 °C for 5 min, followed by 40 cycles at 95 °C for 10 s, 60 °C for 30 s, and 72 °C for 1 s. Relative microbial was performed using the 16S/18S rRNA threshold cycle values for normalization (universal 16S and 18S primers were used for normalization), and using the control group for comparison according to the 2^−ΔΔCq^ method [7]. Species relative abundance was calculated as fold change compared to the healthy control.

The following bacterial populations were investigated: *Actinobacteria*, *Deferribacteres*, *Verrucomicrobia*, *Tenericutes*, *Betaproteobacteria*, *Epsilon proteobacteria*, *Gamma proteobacteria Mucispirrilum*, *Gammatimonadetes*, *Eubacteria*, *Lactobacillus*, BPP, *Clostridium leptum*, *Clostridium cocoides*, *Ruminococcus*, *Firmicutes*, *Bacteroidetes* sp., *F. Prausnitzii*, ARNr 18S, *Saccharomyces*, and *Candida* [8].

#### Statistical Analysis

The data obtained are presented as a mean and standard deviation, and graphs were generated using GraphPad 5 software (Dotmatics).

Differences between groups were determined using the Mann–Whitney test. A *p*-value < 0.05 was considered suggestive of a statistically significant difference.

This study was approved by the Ethics Commission of “NC Paulescu” National Institute of Diabetes, Nutrition and Metabolic Diseases (Bucharest, Romania; approval no. Certif.5911/04.10.2019). All participants gave their written informed consent upon inclusion in this study. This research adhered to the principles outlined in the Declaration of Helsinki and also obtained approval from the Ethics Committee at the University of Bucharest (Bucharest, Romania; under protocol code CEC reg. no. 235/9.10.2019).

### 2.2. The Immunological Study

**The immunological study** had two targets: innate immunity (inflammation) and adaptive immunity (we chose to address the cellular immune response because, unlike the humoral one, it is insufficiently studied in this category of associated pathologies). This part of the study was essential because, although the influence that each of the two pathologies exerts on the body’s immune response is known, their combined effect is much less studied [9].

As markers for innate immunity (inflammation), the following were determined: IL-6 (interleukin 6), sIL-6R (soluble IL-6 receptor), IL-1β (interleukin 1β), TNFα (tumor necrosis factor α), IL-10 (interleukin 10), and NGAL (neutrophil gelatinase-associated lipocalin). TNFβ (tumor necrosis factor β)/LTα (Lymphotoxin α) was determined as a marker for adaptive immunity (cellular immune response).

### 2.3. The Study of Tissue Regeneration Capacity

**The study of tissue regeneration capacity** was performed using NT3 (neurotrophin-3) (this is the first study to do so) and VEGFβ (vascular endothelial growth factor β, another marker that is scarce in this category of patients) as markers.

Venous blood samples were collected from the patients following overnight fasting, aliquoted, and stored at −80 °C until further analysis was performed.

All the aforementioned compounds were determined from serum samples, utilizing Merck Millipore ELISA kits for IL-6, IL-1β, IL-10, NT-3, and VEGF β, and Elabscience ELISA kits for IL-6R, TNFα, TNFβ, and NGAL.

The human ELISA kits utilized in our study were single-wash 90 min sandwich ELISAs designed for the quantitative measurement of human serum, plasma, and cell culture supernatant. SimpleStep ELISA^®^ technology employs capture antibodies conjugated to an affinity tag that is recognized by the monoclonal antibody used to coat the SimpleStep ELISA^®^ plates. This approach to sandwich ELISA allows for the formation of the antibody–analyte sandwich complex in a single step, significantly reducing assay time.

#### Statistical Analysis

For the comparison between groups, the data were processed using the SPSS IBM V26 statistical package, with a statistical significance defined as *p* ≤ 0.05, which is conventionally adopted for checking the degree of data compatibility with the null hypothesis. The statistical protocol was applied according to the design of a cross-sectional study and the type of data collected. Descriptive statistics, such as mean ± standard deviation, median, and range, were used for quantitative variable presentation (such as age, duration of hemodialysis, and serum markers). For comparison between groups (DM patients vs. non-DM patients, IL-6, sIL-6R, etc.), an “independent-samples *t*-test or nonparametric tests (independent-samples Mann–Whitney U test and Kruskal–Wallis test) were applied after the data’s normality distribution was verified. Qualitative variables (such as gender and the presence or absence of DM) were described as structure or intensity indicators (absolute or relative frequencies). The chi-square (X2) test or Fisher’s exact test was also applied to examine the association between the categorical variables. Correlation analysis was also performed. Boxplot graphs (of the minimum, first quartile, median, third quartile, maximum, and outliers of the dataset’s parameters) were created to show the data series’ distribution for the global sample or stratified into subgroups of interest.

For the 82-year-old patient, the results obtained were so different that we could only compare these results with the average levels of the two groups.

All participants gave their informed consent upon inclusion in this study. This study was conducted in accordance with the Declaration of Helsinki and was approved through ethical permit no. 295/17.01.2023.

## 3. Results

### 3.1. The Genetic Study of the Intestinal Microbiota Results

**The genetic study of the intestinal microbiota** did not reveal significant differences in the 82-year-old patient compared with the general results obtained in the test group.

Thus, regarding the major bacterial phyla of the intestinal microbiota—Bacteroidetes and Firmicutes—no significant differences were identified between the two groups (including our patient).

**Figure 1:**
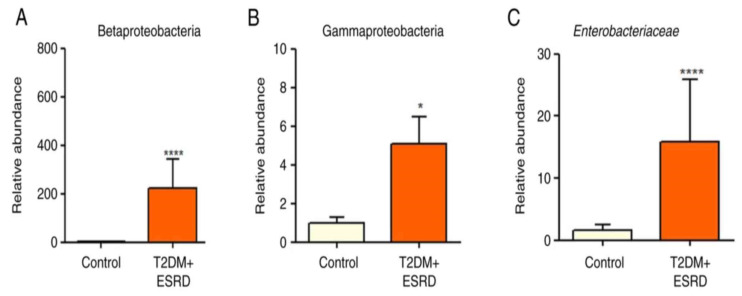
Relative abundance of (**A**) *Betaproteobacteria*, (**B**) *Gammaproteobacteria,* and (**C**) *Enterobacteriaceae* in patients with T2DM and ESRD. * *p* < 0.05, **** *p* < 0.0001 vs. control; Mann–Whitney test. ESRD, end-stage renal disease; T2DM, type 2 diabetes mellitus [8].

The gut microbiota of our chosen patient, along with that of the other patients with T2DM and ESRD, were characterized by increased levels of Gamma proteobacteria. All patients also showed a significantly increased abundance of Enterobacteriaceae. Pre-existing research data associate this microbial population with the existence of an inflammatory process in the large bowel [9,10]. Moreover, high levels of Enterobacteriaceae are considered an indicator of intestinal dysbiosis. In addition, the Beta proteobacteria phylum was significantly more abundant in stool samples from patients with T2DM and ESRD compared to the control group [8] (Figure 1A–C).

**Figure 2:**
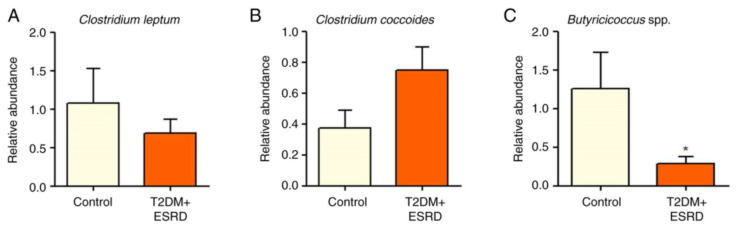
Relative abundance of (**A**) *Clostridium leptum*, (**B**) *Clostridium coccoides,* and (**C**) *Butyricicoccus* in patients with T2DM and ESRD. * *p* < 0.05 vs. control; Mann–Whitney test. ESRD, end-stage renal disease; T2DM, type 2 diabetes mellitus.

The Clostridium genus (represented by *Clostridium leptum* and *Clostridium coccoides*) did not show any significant differences between the two groups of patients (Figure 2A,B).

The abundance of *Butyricicoccus* sp. was also analyzed. This is a bacterial genus with an important role in homeostasis, being involved in the production of short-chain fatty acids (SCFAs), especially butyrate. The intestinal microbiota of the 82-year-old patient, as well as that of all patients with T2DM and ESRD included in this study, presented significantly reduced levels of *Butyricicoccus* (Figure 2C).

There were no significant differences between the group of patients with T2DM and ESRD and the control group with regard to the abundances of *Ruminococcus* sp, Faecalibacterium praustnitzii, and Bacteroides-Prevotella Porphyromonas (BPP).

**Figure 3:**
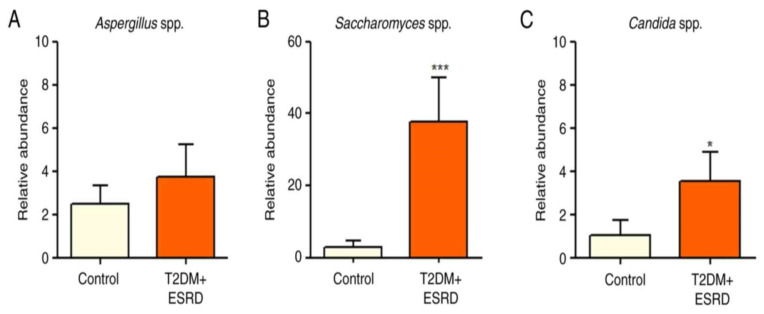
Relative abundance of (**A**) *Aspergillus* spp., (**B**) *Saccharomyces* spp., and (**C**) *Candida* spp. in patients with T2DM and ESRD. * *p* < 0.05, *** *p* < 0.0001 vs. control; Mann–Whitney test. ESRD, end-stage renal disease; T2DM, type 2 diabetes mellitus.

An analysis of the major fungal populations (*Candida* sp., *Aspergillus* sp., and *Saccharomyces* sp.) was also performed [8]. Although no significant differences in *Aspergillus* species were identified between the two groups, both *Candida* and *Saccharomyces* species illustrated significant increases in patients with T2DM and ESRD, including the studied patient (Figure 3A–C).

A series of phyla and microbial populations were not identified by real-time PCR (Ct value > 38) in our patient groups: Mucispirillum, Verrucomicrobia Deferibacteres, and Tenericutes. It is possible that these species present a very low abundance in the observed patients, thus making their identification impossible. In this situation, the aforementioned bacterial populations can only be identified in the future by applying more advanced sequencing techniques.

Conclusions: All the aforementioned intestinal microbiota alterations were also present in the 82-year-old patient who is the subject of this paper, so the patient did not differ from the other members of the test group. Thus, the intestinal microbiota changes cannot justify the spectacular immunological behaviors and tissue regeneration capacity that the patient presented.

### 3.2. The Results of the Immunological Study

The very extensive study of the immunological behavior provided the first extremely surprising results regarding the patient in question, who presented very different values compared to the average of the parameters of the test group, made up of patients with the same pathology. For further clarification, the results were also compared with those of control subjects.

**Figure 4:**
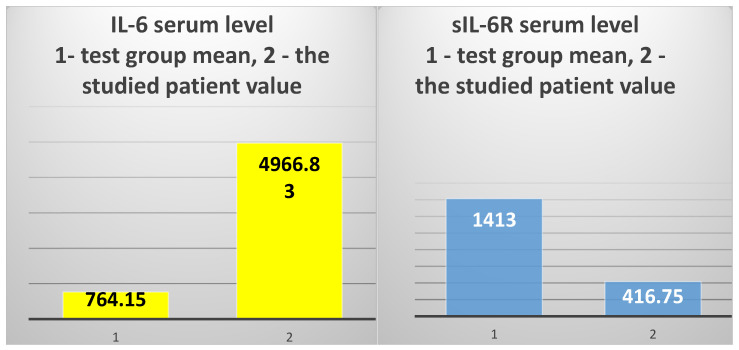
The mean value of IL-6 serum levels in the test group vs. the value obtained in the 82-year-old patient (**left**); the mean value of sIL-6R serum levels in the test group vs. the value obtained in the studied patient (**right**).

The exploration of innate immunity (inflammation) revealed an extremely high IL-6 serum level (4966.83 pg/mL), 6.5 times higher than the average of the test group (T2DM and ESRD) (764.15 pg/mL) and 15.2 times higher than the average of the control group, consisting of ESRD patients (328.38 pg/mL) (Figure 4, left).

However, the serum level of sIL-6R was 3.3 times lower than the average of the test group to which the patient belongs (416.75 ng/mL vs. 1372.94 ng/mL, respectively) and 2.89 times lower than the average of the control group (416.75 ng/mL vs. 1207.84 ng/mL) (Figure 4, right).

**Figure 5:**
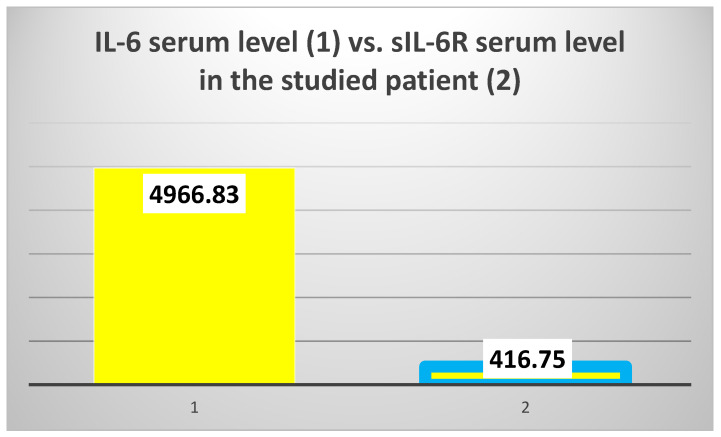
The difference between interleukin 6 (IL-6) and IL-6 soluble receptor (sIL-6R) serum levels.

In addition, the studied patient presented a huge difference between the serum levels of IL-6 and the IL-6 soluble receptor (sIL-6R) (Figure 5).

**Figure 6:**
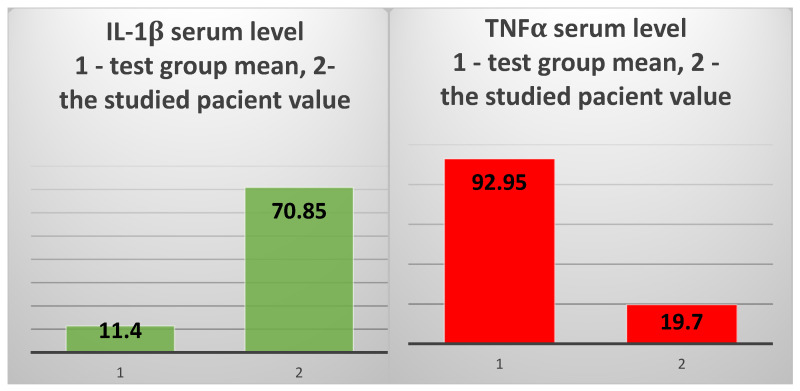
The mean value of IL-1β serum levels in the test group vs. the value obtained in the 82-year-old patient (**left**); the mean value of sIL-6R serum levels in the test group vs. the value obtained in the studied patient (**right**).

The other two inflammatory cytokines, IL1β and TNFα, also showed very different values in the patient compared to the two groups. IL1β had a serum level that was 6.2 times higher than in the test group (70.85 pg/mL vs. 11.4 pg/mL) (Figure 6, left) and approx. 26 times higher than the mean of the control group (70.85 pg/mL vs. 2.73 pg/mL). However, the serum level of TNFα was 5.2 times lower than in the test group (19.7 pg/mL vs. 102.12 pg/mL) and 5.5 times lower than the average of the control group (19.7 pg/mL vs. 108.05 pg/mL) (Figure 6, right).

**Figure 7:**
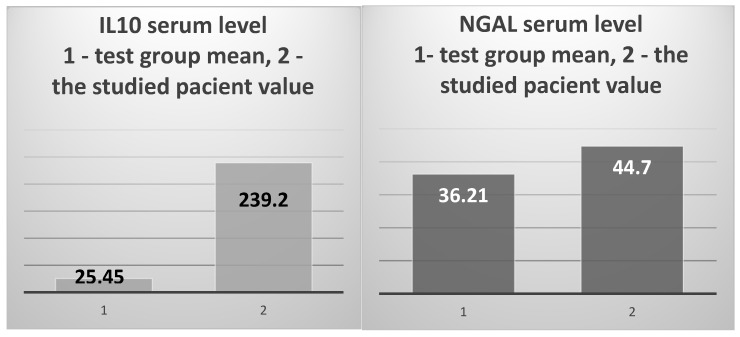
The mean value of IL-10 serum levels in the test group vs. the value obtained in the 82-year-old patient (**left**); the test group’s mean NGAL serum level vs. the value obtained in the studied patient (**right**).

The prototypical anti-inflammatory cytokine IL10 showed a 9.4 times higher level than the average of the test group (239.32 pg/mL vs. 25.45 pg/mL, respectively) (Figure 7, left), and was 10.88 times higher than the average of the control group (239.32 pg/mL vs. 21.99 pg/mL). NGAL showed higher values than the averages of the test group and the control group, without significant differences (44.7 ng/mL vs. 36.21 ng/m and 37.49 ng/mL, respectively) (Figure 7, right).

**Figure 8:**
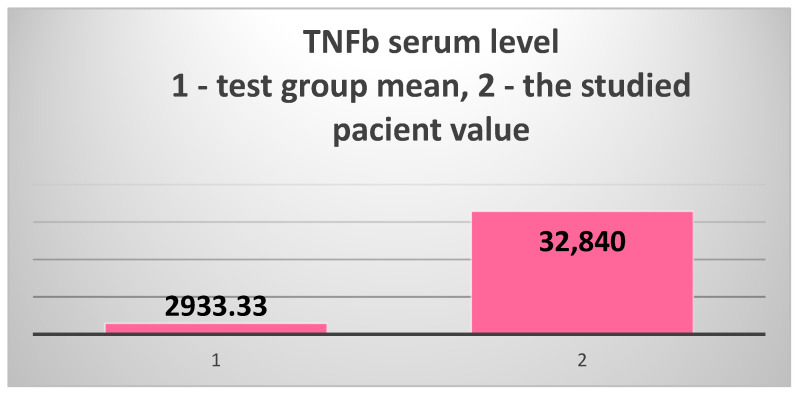
The extremely high serum level of TNFβ (LTα) in the 82-year-old patient vs. the test group’s mean value.

The study of acquired immunity focused on the cellular immune response, which is a much less explored area of study in patients with T2DM and ESRD. We chose the serum level of TNFβ (LTα—lymphotoxin α) as a marker. The results obtained in the patient were again extremely surprising: 32,840 pg/mL, 11.2 times higher than the test group’s average of 2933.33 pg/mL and 25 times higher than the control group’s average of 1308.7 pg/mL (Figure 8).

### 3.3. The Results of Tissue Regeneration Capacity Study

In parallel with the innate and acquired immunity studies, we considered it essential to also evaluate the tissue regeneration capacity of patients presenting with the combination of T2DM with ESRD. As mentioned before, we used the serum levels of NT3 (the first study in these associated pathologies) and VEGFβ (extremely understudied in this category of patients).

The results obtained in the 82-year-old patient were again impressive.

**Figure 9:**
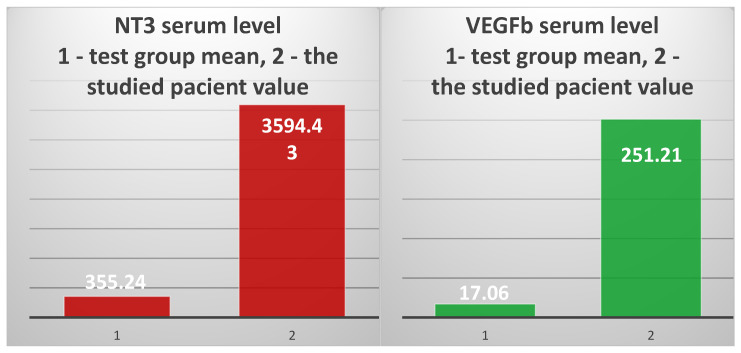
The mean value of NT-3 serum levels in the test group vs. the value obtained in the 82-year-old patient (**left**); the mean of the test group’s VEGFβ serum level vs. the value obtained in the studied patient (**right**).

The serum level of NT-3 was 3594.43 pg/mL, 10.1 times higher than the average of the test group (355.238 pg/mL) and 78.65 times higher than the average of the control group (45.7 pg) (Figure 9, left).

VEGFβ showed a serum level of 251.2 ng/mL, 14.72 times higher than the average of the test group (17.06 ng/mL) and 251 times higher than the average of the control group (1.022 ng/mL) (Figure 9, right).

## 4. Discussion

The 82-year-old patient that this study focuses upon presented three patterns of altered functional behavior: intestinal microbiota pathological alterations, completely unexpected changes in immunological patterns, and tissue regeneration.

### 4.1. The Genetic Analysis of the Gut Microbiota

The genetic analysis of the gut microbiota of the studied patient did not show any differences compared to the other patients with T2DM and ESRD in the test group.

Thus, the patient’s microbiota showed significantly increased levels of Gamma proteobacteria and Enterobacteriaceae [8]. Existing research associates these changes with the existence of an inflammatory process in the colon while also considering these alterations as an indicator of intestinal dysbiosis.

As in the other test group patients, the Beta proteobacteria phylum was significantly more abundant in the patient’s stool samples compared with the control group [8].

The gut microbiota of the test group, including the 82-year-old patient, presented significantly reduced levels of *Butyricicoccus*, a bacterial genus with an important role in homeostasis, being involved in the production of short-chain fatty acids (SCFAs), especially butyrate [8].

The major fungal populations (*Candida* sp, *Aspergillus* sp, and *Saccharomyces* sp.) that were analyzed did not present significant differences between the test and the control groups regarding *Aspergillus* species, but *Candida* and *Saccharomyces* species showed significant increases in T2DM and ESRD, including in the studied patient.

All these changes in patients with T2D and ESRD support the presence of a chronic inflammatory phenomenon at the intestinal, regional, and systemic levels, but they cannot justify the extremely surprising and different immunological behavior and tissue regeneration patterns of the patient this study focuses upon.

### 4.2. The Study of Immunological Pattern

The second functional pattern that was studied was the immunological one, which evidenced both an enhancement of innate immunity (inflammation) and adaptive immunity (intensification of cellular immune response capacity).

#### 4.2.1. The Study of Innate Immunity

The 82-year-old patient presented with an extremely high IL-6 serum level that was 6.5 times higher than the mean of the test group (T2DM with ESRD) and 15.2 times higher than the mean of the control group consisting of non-diabetic hemodialysis patients (ESRD).

Likewise, the other two inflammatory cytokines, IL-1β and TNFα, presented very different values compared with the average of the two groups. Thus, IL-1β had a serum level 6.2 times higher than in the test group and approx. 26 times higher than the mean of the control group. However, TNFα showed concentrations 5.2 times lower than the test group and 5.5 times lower than the average of the control group.

Let us try to understand these alterations.

At first sight, the results prove the presence of a much more intense inflammatory reaction in comparison with both the test group and the control group, which seems to be plausible in an 82-year-old patient with multiple associated pathologies. However, although the IL-6 and IL1β serum levels were much higher than those of both patient groups’ mean values, the level of TNFα, a powerful pro-inflammatory cytokine, was much lower. How can this decrease be explained?

Another very interesting aspect concerns the IL-6 soluble receptor (sIL6R). The patient presented an sIL-6R serum level 3.3 times lower than the mean of the test group to which she belongs and 2.89 times lower than the control group’s mean value, even though the IL-6 serum level was high.

These differences are difficult to explain without prior knowledge of the functional and informational interferences between inflammatory cytokines.

a. IL-6 is the prototypical cytokine of a large family of signal molecules, which also include IL-11, LIF (leukemia inhibitory factor), OSM (oncostatin M), CNTF (ciliary inhibitory factor), CT-1 (cardiotrophin 1), NNT-1 (cardiotrophin-like related cytokine and stimulating neurotrophin1/B-cell stimulating factor 3), NPN (neuropoietin), IL-27, and IL-31 [11].

IL6 is a signal molecule synthesized by many cell types: B cells, T cells, macrophages (including microglia), dendritic cells, mast cells, vascular endothelial cells, fibroblasts, keratinocytes, and mesangial cells [12]. It is important to note that mesangial cells are a source of IL-6. These cells are contractile cells that constitute the central stalk of the glomerulus [13]. They are stromal cells with an important role in maintaining glomerular homeostasis and responses to aggression. They are involved in inflammation, immune responses, regeneration, and fibrosis [14].

IL-6 synthesis is stimulated by IL-1β, TNFα, PAMPs and DAMPs, TLR (toll-like receptor) activation, prostaglandins, adipokines, and other cytokines. Currently, it is known that IL-6 signaling is carried out through three pathways: through fixed membrane receptors (IL-6R or mIL-6R), through soluble receptors (sIL-6R), and through “trans-presentation”, a recently described pathway.

IL-6R membrane receptors are present on immune cells and hepatocytes. For signal transduction, the ubiquitous transmembrane glycoprotein gp130 is used. All IL-6 family cytokines except IL-31 use gp130 for signal transduction. Thus, the binding of IL-6 on the IL-6R results in the attachment of gp130, forming a heterotrimer that then dimerizes [15]. A hexameric complex is formed that triggers transduction through phosphorylation cascades, with the JAK family tyrosine kinases (Janus kinases) being the first activated. The result is the activation of STAT transcription factors (signal transducers and activators of transcription) [16].

The second signaling pathway, known as trans-signaling, involves the binding of IL-6 to the soluble receptor sIL6R. This is achieved either by alternative mRNA splicing or by membrane IL-6R cleavage by different proteases, the best-known being the ADAM17 metalloprotease [17]. Any inflammatory reaction is an important source of proteases and, therefore, sIl6-R. The IL6-sIL6R complex binds to gp130, which is present in all cellular membranes, and generates signal transduction. Thus, IL-6 can stimulate all cells, even if they lack a membrane Il-6R.

What is very interesting and important to note is that trans-signaling is pro-inflammatory, whereas classic IL-6 signaling through the membrane IL-6R is pro-regenerative and anti-inflammatory [18,19].

The third pathway, also called trans-presentation, was identified at the level of dendritic cells, which are known to play a role as APCs (antigen-presenting cells). These cells present the IL6-sIL6R complex to TH (CD4+) lymphocytes, which fix it upon the membrane gp130 and, secondary to transduction, transform into TH17 lymphocytes, secreting IL-17, with a strong pro-inflammatory role and favoring autoimmunity [19].

The fact that our patient’s sIL-6R serum level was approximately 3-fold lower than the mean value of the test group in which she is included, while her IL-6 level was 6.5 times higher, enables us to say that, in this case, IL-6 predominantly exerts anti-inflammatory and pro-regenerative actions, mediated by the membrane IL-6R.

However, in an attempt to understand the complexity of the informational interference between the parameters studied in the 82-year-old patient, it was mandatory to compare the serum IL-6 changes not only to the level of the sIL-6R but also to the results obtained for the other two major pro-inflammatory cytokines, IL-1β and TNFα, and for the prototypic anti-inflammatory cytokine IL-10.

It is known that IL-6 exerts an inhibitory effect on TNF synthesis, not at the brain level but in the periphery [20].

The inhibitory effect of IL-6 on IL-1 synthesis is also known [21].

Let us see if these influences are also expressed in our results.

b. The serum level of IL-1β also offered surprises. Thus, we obtained serum levels that were approximately 6-fold higher than the mean of the test group (T2DM) and 26 times higher than the mean value of the control group (ESRD).

IL-1β is produced by leukocytes, particularly monocytes and macrophages, and is responsible for a multitude of biological actions:

- fever induction [22,23,24,25];
- stimulates neutrophilia and acute-phase protein discharge [26,27];
- stimulates mitosis of antigen-induced T cells and B cells [28,29,30];
- stimulates the synthesis of arachidonic acid metabolites and the release of chondrocytes and other cell types from fibroblasts [31];
- stimulates the synthesis of chemokines (IL-8 and MCP-1) and inflammatory cytokines (autocrine and paracrine stimulation of its own synthesis, IL-6, and TNFα) [32,33];
- stimulates the synthesis of reactive oxygen and nitrogen species [32,33];
- regulates glucose and corticosteroid homeostasis [32,33];
- stimulates humoral and cellular immune responses by stimulating helper lymphocytes and TH1, TH2, TH17, and CD8+ cytotoxic T lymphocytes [34,35,36].

IL-1 does not involve only one molecule type but a superfamily consisting of IL-1β and eight other cytokines derived from IL-1β (IL-1α, IL-1Ra, IL-36Ra, IL-36Rα, IL-36Rβ, IL-36Rγ, IL-37, and IL-38) [37,38]. IL-18 and IL-33 are also included due to their structural and functional similarity to IL-1 [39].

The IL-1 cytokine family exerts its actions through the IL-1R receptors, which represent another molecular family consisting of ten types of transmembrane proteins. IL-1Rs have an extracellular region consisting of three Ig-like domains, and an intracellular region containing a TIR domain (Toll-IL-1R), which is identical to that of a TLR (toll-like receptor [40]. The prototypical and most studied receptor is IL-1R1, which binds IL-1β and IL-1α.

There is also an IL-1R antagonist, IL-1Ra, which blocks the binding of IL-1β and IL-1α to the IL-1R.

What is very interesting is that the IL-1 molecular family can exert certain actions without binding to the IL-1R. This aspect has been proven in cerebral ischemic lesions [41].

The explanation of this phenomenon is based upon the existence of a lectin-like domain in the IL-1β molecule, through which the cytokine can bind to carbohydrates in the target cell membrane (heparin, hyaluronic acid, and GM4) and in the extracellular matrix. It is a way to short-circuit binding on the IL-1R [42,43,44,45,46].

Another extremely important finding is the evidence of increased serum levels of IL-1β in patients with chronic hypoxia, with levels correlating to disease severity. The trigger mechanism of inflammation involved in this situation is the activation of lysosomal enzymes in hypoxic cells by metabolic acidosis secondary to exacerbated glycolysis. The first favorable effect of the inflammatory reaction is local vasodilatation, with partial or total correction of hypoxia.

Our patient has secondary diagnoses of HF class III NYHA with vascular substrate and ischemic coronary disease, so she presents a certain degree of myocardial and tissue hypoxia. This level of hypoxia is also accentuated by diabetic arteriopathy.

The conditions for an increase in the serum level of IL-1β are therefore met.

Recent studies on human cardiac fibroblasts isolated from patients with heart failure have shown that these cells become immunocompetent through the appearance of CD4 receptors on their membranes [47].

The underlying mechanism is the stimulation of CD4 expression in cardiac fibroblast membranes by IL-1β. Thus, fibroblasts acquire phenotypic and functional characteristics similar to T helper cells (TH) and T reg CD4+ cells (approx. 10% of suppressor T lymphocytes). There are research papers that show that IL-1β acts as a growth factor for intestinal and renal fibroblasts, but also as a regulator of collagenase expression [48,49].

Experimental cardiac fibroblast stimulation with IL-1β resulted in the increased synthesis of more than 300 proteins. Among them, IL-8, IL-10, and MCP-3 showed a massive increase. Secretion of IL-6, IL-12p70, TNFβ, and VEGFβ was increased, but not significantly [50].

Existing research data therefore suggest that pro-inflammatory stimuli rapidly stimulate cardiac fibroblasts, as well as intestinal and renal fibroblasts, to genetically and phenotypically reprogram into immune cells that amplify inflammation and recruit other immune cells.

The intracellular signaling pathways by which fibroblasts execute this reprogramming are not known.

However, a fundamental question arises: do fibroblasts transform into TH CD4+ or TregCD4+ (suppressor) lymphocytes?

Corroborating all the data obtained, we can state that in the studied 82-year-old patient, there are greater chances of directing fibroblasts toward the LTregCD4+ profile.

c. TNFα was also a major surprise. The patient’s TNFα serum level was 5.2 times lower than the test group’s mean value and 5.5 times lower than the control group’s. We must note that the mean value of the test group (T2DM+ESRD) was close to that of the control group (ESRD). The initial, intermediary conclusion is that the presence of T2DM does not amplify TNFα’s pro-inflammatory effect.

However, how can one explain the great difference between the serum levels of the two pro-inflammatory cytokines (IL-6 and TNFα)?

It is known that TNFα is initially synthesized in the RER (rough endoplasmic reticulum) as a 26 kDa protein (233 amino acids) that remains transmembrane-enclosed (mTNF). Part of this TNF remains as it is, while another part is cleaved by a TNF-converting enzyme into a soluble fragment of 17 kDa and 157 amino acids (sTNF). Both molecules exert their action at the cellular level through TNFR1 and TNFR2 receptors. TNFR1 is present on all cell types, whereas TNFR2 is found only on the membrane of immune cells, endothelial cells, and neurons [51,52,53,54].

A membrane TNF molecule (mTNF) is a ligand for another cell’s membrane TNFR, creating a connection that constitutes a method of intercellular informational exchange. TNFR2 is the receptor that binds to mTNF with the highest affinity, inducing a stronger cellular response than sTNF [53].

Because TNFR2′s intracellular segment is very short, it needs TRAF molecules (TNF receptor-associated factors) for signal transduction. More precisely, TRAF1 and TRAF2 molecules are required. These were originally considered adapter proteins. In addition to TNFR2, they interact with the intracellular domains of TLRs (toll-like receptors), TCR (LT antigen recognition receptor), TGFβR, interferon receptors, interleukin receptors (including IL-6), and platelet receptors [54].

These TRAF molecules control kinases involved in receptor-triggered signal transduction, and their structural and functional alterations induce various pathologies.

The intracellular segment of TNFR1 associates with a death domain (DD), thus making this receptor capable of inducing both cellular survival and cell death through extrinsic apoptosis.

A very important thing to note is that the intracellular segment of TNFR2 does not attach to the DD domain, so it is only involved in inducing the survival of the immune cell, endothelial cell, and neuron.

The 82-year-old patient, who is the focus of this study, presented a significantly lower level of soluble TNFα compared with the mean value of the test group in which she was included.

We can hypothesize that in the case of this patient, the pro-regenerative effect of the transmembrane form, mTNF, exerted through TNFR2 is the one that predominates. We are justified in doing so because the behavior of IL-6 also confirms the same scenario and because the regeneration markers NT-3 and VEGFβ presented very high serum levels.

d. The serum level of IL10, the prototypical anti-inflammatory cytokine, was 9.4 times higher than the test group’s mean and 10.88 times higher than the average of the control group.

When analyzing the studied cytokines comparatively, we can conclude that the 82-year-old patient with T2DM and ESRD showed the highest increase for IL-10 compared with the other patients of the group in which she was included, as well as compared with the serum levels obtained for the other cytokines. This is an extremely interesting new aspect that needs to be understood.

IL-10 is secreted by multiple cell types: TH1, TH2, and TH17 cells, Tregs, lymphocytes, dendritic cells, monocytes and macrophages [55,56], microglia and cardiac macrophages [57,58], B cells, mast cells, NK cells, and eosinophils [59].

Cytotoxic T cells (CD8+) also become an important source of IL-10 during hypoxia and viral infections [60].

In addition to all these sources of IL-10, epithelial cells and fibroblasts in the intestine, as well as skin keratinocytes, secrete IL-10 when infected or subjected to UV radiation, aggression, and tissue damage [61,62,63,64]. Even certain malignant cells (melanoma, colon, and breast carcinoma) secrete IL-10 [65,66,67,68].

In contrast to all these cell types, human neutrophils do not produce IL-10, even when stimulated by bacterial or pro-inflammatory molecules [69].

IL-10 belongs to class II cytokines and is a soluble homodimer of 36 kDa in its active form [70]. Each monomer has six α-helices and is linked to the other by two disulfide bonds [71].

Cellular responses are mediated by the binding of IL-10 to the IL-10R receptor, a tetrameric receptor belonging to the IFN receptor (IFNR) family [72,73]. It has two IL-10 binding subunits, called IL-10R alpha (IL-10RA), and two accessory signal transducing subunits called IL-10 beta (IL-10RB) [72,73]. IL-10RA has a high affinity for IL-10, in contrast to IL-10RB, which has low or no affinity [58]. IL-10RA is the constitutive receptor of lymphocytes, macrophages, and dendritic cells, but the number of receptors increases significantly after cellular activation [74].

Although it has a low affinity for IL-10, IL-10RB is present on almost all cell membranes and is also used in IL-22 and IL-26 receptor complexes [75].

IL-10 signaling first involves the oligomerization of IL-10RA with IL-10RB, then the activation of some tyrosine kinase cascades, and finally the activation of the STAT3 transcription factor, which enters the genome [76].

Although long considered the prototypical anti-inflammatory cytokine, it is currently known that IL-10 exerts dual effects: anti-inflammatory and immunosuppressive. On the other hand, it also possesses immunostimulatory properties [77,78].

However, IL-10 secretion begins late in viral, bacterial, fungal, and parasitic infections, when the PAMPs, DAMPs, and native Ag amounts are greatly reduced [79,80].

It should not be overlooked that certain infections stimulate IL-10 synthesis in order to ensure that the pathogen survives in the host organism [81].

Having said that, let us systematize the anti-inflammatory and immunosuppressive actions of IL-10:

- Inhibition of gene reading for pro-inflammatory proteins in monocytes, macrophages, and dendritic cells, with the consequent inhibition of IL-1β, IL-1α, TNFα, IL-6, IL-12, and IL-18 secretion, as well as of chemokines CCL2, CCL5, CCL12, IL-8, CXCL2, and CXCL10 [58];
- Stimulates the synthesis of the IL-1R antagonist (IL-1Ra), which blocks IL-1β signaling [82];
- Inhibits the reading of IL-4 and IL-13 genes in monocytes and dendritic cells [82,83];
- Inhibits IL-2 and IFNγ release from TH1 cells [84];
- Suppresses the ability of APCs to present MHCII-epitope complexes to TH1 and TH2 cells [85];
- Inhibits the expression of the intercellular adhesion molecule ICAM-1, the ligand CD80 (B7), and the CD 56 receptor on the APC membrane, thus inhibiting the formation of the immune synapse between the APC and T cells (TH and cytotoxic T cells, respectively) and the complete activation of T cells [86,87,88];
- Inhibits activation of CD4+ lymphocytes through TCR receptors and the consecutive secretion of IL-2, IL-4, IL-5, IFNγ, and TNFα [89,90];
- Inhibits the cytotoxic activity of T cells [89,90];
- Blocks T cell stimulation by its co-receptor CD28 [91,92].

The immunostimulatory activities of IL-10 are as follows:

- It strongly stimulates B lymphocytes to pass in the intermediate stage of activation, i.e., to become B lymphoblasts (BLb);
- Inhibits BLb apoptosis and stimulates their clonal growth and expansion, as well as the full activation of BLb in immunoglobulin-secreting plasma cells (plasmocytes) [93,94];
- Stimulates the differentiation and activation of cells that are already committed to apoptosis to T reg cells, with their functional recovery [95,96,97,98];
- Stimulates the proliferation of thymic cells [99];
- Stimulates CD8+T cells (cytotoxic T lymphocytes) by increasing their expression of MHCI molecules and the secretion of IFNγ and granzymes [100];
- Stimulates NK proliferation and migration, as well as their cytotoxic functions [101,102,103,104];
- Stimulates mast cells [105].

A very important action of IL-10, which must also be corroborated with the data obtained for NT-3 in our study, is exerted on CNS and peripheral neurons [57,59,106]:

- Reduces neuronal injury during infections, inflammation, ischemia, and trauma;
- Increases the lifespan of neurons and axonal regeneration;
- Modulates neurogenesis in adults.

IL-10 also stimulates the repair of the colonic epithelium and the post-injury regeneration of the dermis and endothelium [107].

#### 4.2.2. The Study of Acquired Immunity

The general study on acquired immunity that we realized focused on the cellular immune response (cell-mediated immunity), a much less explored area in patients with T2DM and ESRD.

We chose TNFβ as a marker, which has been scarcely explored, for which the serum level was measured. The result obtained in the 82-year-old patient was again extremely surprising: her TNFβ serum level was approx. 11 times higher than the test group’s mean value and 25 times higher than the control group’s. **The patient’s serum TNFβ level was 1667 times higher than that of TNFα**.

How can we explain this behavior in the context of general changes in immunological and tissue regeneration patterns?

TNFβ, also known as lymphotoxin α (LTα), is a cytokine that has 30% homology to TNFα in terms of its primary structure and a much more pronounced similarity in its tertiary and quaternary structures [108].

LTα is a soluble homotrimer (three monomers of 17 kDa) that exerts its biological effects using TNFR1 and TNFR2 receptors, as does TNFα.

Currently, the known multiple cellular sources of LTα are antigen- and mitogen-activated T lymphocytes [109], B lymphoblasts [110,111], NK cells and macrophages [112], microglia, astrocytes, and neurons [113]. LTα secretion from microglia is stimulated by IL-12, IL-16, and bacterial LPS, but TNFα and IFNγ have no effect [114].

LTα exhibits a unique functional characteristic among TNF family members: it is never found as a single unit on the cell membrane, although it binds with high affinity to TNFR1 and TNFR2 [115]. Its membrane binding occurs only in a complex with membrane lymphotoxin β (LTβ) [116]. A heterotrimer consisting predominantly of one LTα molecule and two LTβ molecules (LTα1β2), with LTα2β1 being less common, is formed.

The LTαβ heterotrimer binds to the transmembrane receptor LTβR, and the result of the triggered phosphorylation cascades is the activation of the NF-κB transcription factor.

The currently known functions of TNFβ/LTα can be systematized as follows:

- stimulates the development of lymphoid organs. It is experimentally proven that TNFβ stimulates the formation of intestinal Payer’s plaques through TNFR1 [117];
- stimulates the cellular immune response (the cytotoxic and tumoricidal actions of cytotoxic T CD8+ cells and NK lymphocytes, as well as the cytotoxic actions of macrophages) [118,119,120].

In addition to its cytotoxic and tumoricidal actions, the direct neurotoxic effect of LTα has also been proven through the induction of neuronal apoptosis via the extrinsic pathway [121]. Another very important aspect that has been experimentally confirmed is the secretion of LTα by T cell clones that attack myelin basic protein [122].

The stimulation of cellular immunity underlies defenses against Staphylococcus aureus [123], Mycobacterium, Leishmania, and Plasmodium [124,125,126,127].

This induces and stimulates RIC-independent and associated inflammatory reactions [119] without the need for LTβ co-participation in membrane signaling [128].

The mechanisms by which LTα induces inflammation are not clearly proven, but the following possibilities are discussed:

- Stimulation of the expression of intercellular adhesion molecules (ICAMs) and E-selectin on endothelial cell membranes [129]. This aspect has been clearly demonstrated for the endothelial cells of the inflamed pancreas and kidney [130] and is of major importance for our study addressing the associated pathologies of these two organs. Furthermore, this exposure of adhesion molecules was shown to be independent of cytokines secreted by T and B cells;
- LTα induces the expression of the chemokines RANTES (regulated upon activation normal T cell expressed and secreted) and MCP-1 (monocyte chemoattractant protein 1) on murine endothelial cells [131];
- Stimulates the synthesis of pro-inflammatory cytokines from macrophages at the same level as TNFα [132];
- Stimulates inflammatory lymphangiogenesis [133].

All the obtained data regarding TNFβ entitle us to state that the 82-year-old patient studied in this paper presents a strong activation of cellular cytotoxicity and tumoricidal capacity, without existing personal clinical and paraclinical data indicating any suspicion in these pathophysiological directions. Of course, the background pathology raises the need for strong cellular immunity, but it is surprising how, at this age and without acute pathologies to justify it, there is such a great fighting capacity of the body.

### 4.3. Tissue Regeneration Capacity in Patients with T2DM and ESRD

This study was performed by determining the serum levels of NT-3 and VEGFβ. We chose these parameters because degenerative changes induced by diabetic arteriopathy and neuropathy are undoubtedly present. These results were also compared with those of the control group (ESRD patients without T2DM). Is important to note that the NT3 study is a first for these categories of patients, and VEGFβ has been extremely under-analyzed up to this point [4].

a. Overall, results showed decreased values of serum NT-3 and VEGFβ levels in both groups. However, the mean serum level of NT-3 was approx. eight times higher, and that of VEGFβ was about seventeen times higher in the test group compared to the control group [4]. This proves the necessity of increased tissue regeneration in diabetes patients.

The 82-year-old patient again presented unexpected NT-3 serum level values: approx. 10 times higher than the test group’s mean value and about 79 times higher than the mean of the control group. The preliminary conclusion from the study we conducted on the two pathologies is that T2DM induces a much more intense neuroregenerative response than ESRD. However, the serum NT-3 level of the patient presented in this paper exceeded all expectations.

NT-3 was the third discovered neurotrophic factor, after nerve growth factor (NGF) and brain-derived neurotrophic factor (BDNF), and is secreted in the brain, heart, and liver, but also in the pancreas and kidneys [134].

It is a small protein with a molecular weight of 13.6 kDa that shows structural similarities to NGF and BDNF [135]. Its distribution and influence on different neuronal populations clearly differentiate NT-3 from NGF and BDNF. Thus, to achieve its actions, it binds with high affinity to the tyrosine kinase receptor TrkC and with low affinity to the receptors TrkA, TrkB, and p75^NTR^.

Upon NT-3 binding, TrkC dimerizes and autophosphorylates on the tyrosine residues of its intracellular segment. Adaptor proteins will be fixed on these residues, to which the PLCγ enzyme will initially be added, then the PI3K/Akt, MAPK/ERK, and JNK/c-Jun kinase cascades will be triggered. The most important signals for neuronal regeneration and inhibition of apoptosis are transmitted through the PI3K/Akt pathway [136].

There are also inactive forms of TrkC, which lack a tyrosine kinase domain but compete with functional TrkC receptors for NT-3 binding. The result of their action will be a decrease in NT-3 bioavailability and in normal TrkC activation [137].

Through the mentioned pathways, NT-3 regulates neuronal morphology, both in the CNS and in the peripheral nervous system, and both in vivo and in vitro. It has an important role in neural embryogenesis and in the differentiation and development of nerve cells. On adult neurons, it exerts a role of functional regulation and repair after various forms of aggression.

In vitro studies have shown that NT-3 maintains the survival of sympathetic neurons, sensory and motor neurons in the cerebral cortex, motor neurons in the anterior horns of the spinal cord, and acetylcholine-secreting neurons in the basal forebrain and the mesopontine tegmentum area [138].

The same actions of stimulating neuronal proliferation and differentiation, as well as modulating the action of other neurotrophic factors, were also confirmed for NT-3 in vivo.

NT-3 has recently been found to have immunomodulatory effects on both normal immune cells and immune cells derived from ischemic stroke patients. Thus, NT-3 has been shown to attenuate the immune responses generated in this category of patients by decreasing the activation of monocytes, T CD4+ cells, and T CD8+ cells, as well as their cytokine production. The survival time of these cells was not affected. Most likely, NT-3 activates immune system cells using the TrkC receptor signaling pathway, as there was an increase in the number of these receptors [139].

As a preliminary conclusion, we can state that NT-3 induces neuronal survival both by direct effects and by decreasing the intensity of innate and adaptive immunity reactions.

On the other hand, inflammatory cytokines exert mixed effects on the actions of nerve growth factors. The negative influences of IL-1β on the regenerative capacity of nervous tissue by blocking the action of neurotrophins are known [140].

Knowing that neurotrophins stimulate the growth and plasticity of axons, the effect of IL-1β on this phenomenon was investigated, with researchers estimating a negative influence. Thus, they used cultures of neurons from the brain and spinal cord and analyzed the influence of the neurotrophins NGF, BDNF, NT-3, and NT-4 on the density and length of neurites. It was observed that only NT-3 generated a significant increase in the density and length of neuronal extensions. The big surprise was provided by IL-1β, which showed a similar effect to NT-3. Moreover, the simultaneous administration of IL-1β and NT-3 induced a much stronger stimulation of the growth of neuronal extensions than the two factors taken separately [140].

The studied patient presented a serum IL-1β level 6 times higher than the test group’s mean value. Together with the increase in the serum level of NT-3, it may indicate a marked pro-regenerative action at the neuronal level. Another very important aspect is the 20-fold increase in the mean level of IL-1β in the test group (T2DM and ESRD patients) compared to the control group mean (only ESRD patients). Added to this change is an almost 8-fold increase in serum NT-3 levels in the test group compared to the control group’s mean. Through the common behavior of IL-1β and NT-3, we can conclude a very high need for neuroregeneration in the group with T2DM.

IL-1β also stimulates IL-6, TNFα, and nitric oxide (NO) synthesis, as well as the proliferation of macrophages and astrocytes [141,142,143,144]. In turn, both TNFα and IL-6 stimulate the growth of neuron extensions [145,146].

IL-1β stimulates astrocytes to synthesize NGF and FGF, which, in turn, stimulate the regeneration of adjacent injured neurons [147,148,149].

IL-6 is also a potent stimulator of the growth of neuronal extensions and the regenerative capacity of neurons. Evidence is provided by the synthesis of IL-6 from neurons, microglia, astrocytes, epithelial cells, and macrophages both in the CNS and the peripheral nervous system (PNS), as well as the information exchange between the IL-6/gp130 signaling pathway and the neurotrophin signaling pathway [150].

In the case presented by us, the decrease in serum concentrations of sIL-6R, the receptor through which IL-6 exerts pro-inflammatory and anti-regenerative effects, is clearly proven. Therefore, the very high serum level of IL-6 signals primarily through fixed membrane receptors, generating an anti-inflammatory and pro-regenerative effect.

Corroborating the results obtained in the studied 82-year-old patient, we can state that the main co-stimulatory factors of the neuroregenerative action exerted by NT-3 in the central and peripheral nervous systems are IL-6 and IL-1β. The important contribution of IL-10, identified in very high concentrations, is also added. This cytokine is also shown to act on central and peripheral neurons, reducing injury during infection, inflammation, ischemia, and trauma and increasing neuronal lifespan and axonal regeneration [57,59,106].

b. Because both chronic hemodialysis and diabetic angiopathy predispose to the occurrence of vascular and tissue damage, we also monitored the regeneration capacity of these structures in the hemodialysis patients included in this study.

Angiogenesis is a multistep process initiated by growth factors and cytokines. Two categories of such factors are very well-characterized [151]:

(1) some that have direct angiogenic actions, such as vascular endothelial growth factor (VEGF) and fibroblastic growth factor (acidic (aFGF/FGF1) and basic (bFGF/FGF2));
(2) others that have indirect angiogenic actions, such as TNFα and transforming growth factor β (TGF-β).

Among the VEGF family members, VEGFα has been shown to have a strong role in the vascular system, both in inflammation and in angiogenesis [152].

We set out to study VEGFβ, which has a biological action that is much less clarified both in general and especially in hemodialysis patients (extremely few studies).

VEGF-β was initially considered to have an angiogenic role, along with VEGF-α, because of their high sequence homology and similar receptor binding patterns. Current evidence suggests that VEGF-β has a more nuanced role in angiogenesis than VEGF-α [153].

VEGF-β inhibits the apoptosis of several cell types in the vascular wall (endothelial cells, pericytes, and smooth muscle cells) in pro-degenerative conditions, while in the presence of high levels of angiogenic growth factors, it acts as their inhibitor. In this way, VEGFβ ensures the normal vessel density of tissues [154].

Some authors believe that VEGFβ potentiates angiogenesis by increasing the bioavailability of VEGFα [143], but most agree that VEGFβ alone cannot initiate angiogenesis and an increase in vascular permeability [155,156].

On the other hand, VEGFβ is also involved in the informational exchange between endothelial cells and preadipocytes, which takes place in both angiogenesis and adipogenesis [157,158].

Another extremely interesting and important aspect is that VEGFβ exerts actions on many cell types, including on three types of vascular cells (endothelial cells, pericytes, and smooth muscle cells), different types of neurons, and cardiac myocytes. Most of these actions are exerted through the tyrosine kinase receptor VEGFR-1 and its co-receptor NP-1 (neuropilin-1) and involve the inhibition of apoptosis and therefore cell survival [159]. Thus, VEGFβ stimulates the survival of neurons in the cerebral cortex and retina, and motor neurons in the spinal cord [153,160].

We can conclude that VEGFβ stimulates the survival of the mentioned cell types much more strongly than it stimulates angiogenesis.

As our study obtained a serum level of VEGFβ that was approximately 17 times higher than in non-diabetic hemodialysis patients, we considered that diabetic hemodialysis patients can better tolerate the presence of pro-degenerative conditions (VEGFβ exerts an inhibitory effect on the apoptosis of vascular cells, neurons, and myocardiocytes) [4].

The 82-year-old patient again broke away from the general pattern, with VEGFβ serum levels approximately 15 times higher than the average of the test group and 251 times higher than the average of the control group. Correlating this pattern with that obtained for NT-3, we can assume that the regenerative signals are primarily addressed to neurons affected by diabetic neuropathy.

## 5. Conclusions

The most difficult aspects to explain in this elderly patient are the extremely elevated serum levels of IL-1β, IL-6, IL-10, AND TNF-β, as well as of the tissue regeneration markers NT-3 and VEGFβ, compared to all the other members of the group to which she belongs.

A technical error or the poor quality of the ELISA kits cannot be incriminated because the values for all the studied parameters were extremely different from the rest of the test group only in this patient.

All the clinical data exclude the presence of an infection or acute inflammation, which would justify the increase of pro-inflammatory cytokines.

IL-10, the prototype of anti-inflammatory cytokines, also showed a much greater increase (over 9-fold compared to the test group’s mean) than the pro-inflammatory cytokines IL-1β and IL-6 (both increased by approx. 6-fold). Therefore, there is control over the inflammatory reaction.

Moreover, the significant decrease in sIL-6R serum levels corroborated the important increase of IL-6 and points towards a preponderance of the anti-inflammatory and pro-regenerative effects that IL-6 exerts and which are added to those of IL-10.

We must also add the serum behavior of TNFα, which was 5.5 times lower than the test group’s mean. We hypothesize that IL-6 inhibits TNFα synthesis and that, in this patient, the pro-regenerative effect of the transmembrane form mTNF, exerted through TNFR2, predominates. We are justified in doing this because the behavior of IL-6 confirms the same scenario, and the regeneration markers NT-3 and VEGFβ showed very high levels.

On the other hand, the acquired immunity study revealed a very large increase in TNFβ/LTα serum levels compared to the test group’s mean (more than 11 times). This allows us to state that the 82-year-old patient presents a strong activation of cellular cytotoxicity and tumoricidal capacity, without existing personal clinical and paraclinical data indicating any suspicion in these pathophysiological directions. Of course, the background pathology raises the need for strong cellular immunity, but it is surprising how, at this age and without acute pathologies to justify it, there is such great fighting capacity of the body through cellular immunity.

The changes presented above are convergent with the great increase in tissue regeneration capacity, mainly neuronal, highlighted by NT-3 and VEGFβ.

Is there a constitutional or post-infectious acquired genetic component to cytokine overproduction? Is this only for IL-1β, IL-6, and IL-10? How is it possible that the TNFα level is significantly low while the TNFβ level is very high?

How can the great capacity for tissue regeneration be explained?

All the data obtained by us, corroborated with those from the scientific literature, entitle us to hypothesize that the causal element of these behaviors is genetically conditioned IL-6 overproduction (possibly acquired post-infection). Against this background, an 82-year-old patient with T2DM and ESRD, on hemodialysis and with Parkinsonian syndrome, is lucid and within functional limits.

## Data Availability

The authors consent to data sharing in a publicly accessible repository.
